# Body Mass Index and Waist Circumference as Determinants of Hemostatic Factors in Participants of a Population-Based Study

**DOI:** 10.3390/medicina59020228

**Published:** 2023-01-26

**Authors:** Maximilian Iglesias Morcillo, Dennis Freuer, Annette Peters, Margit Heier, Christine Meisinger, Jakob Linseisen

**Affiliations:** 1 Chair of Epidemiology, University of Augsburg, 86156 Augsburg, Germany; 2 Institute of Epidemiology, Helmholtz Zentrum München, 85764 Neuherberg, Germany; 3 Chair of Epidemiology, Institute for Medical Information Processing, Biometry and Epidemiology, Medical Faculty, Ludwig-Maximilians-Universität München, 81377 München, Germany; 4 KORA Study Centre, University Hospital Augsburg, 86156 Augsburg, Germany; 5 Institute for Medical Information Processing, Biometry and Epidemiology, Ludwig-Maximilians-Universität München, 81377 München, Germany

**Keywords:** Body Mass Index, waist circumference, coagulation, hemostatic factors, obesity, KORA

## Abstract

*Background*: In contrast to studies in patients, an association between obesity and blood coagulation factors has not been established in the population. If confirmed it could become a target for primary prevention. *Objective*: To investigate the relationship between Body Mass Index (BMI) and waist circumference (WC) with plasma concentrations of antithrombin III, D-dimers, fibrinogen D, protein S, factor VIII, activated partial thromboplastin time (aPTT), quick value, and international normalized ratio (INR) in the general population. Materials and *Methods*: Participants of the Cooperative Health Research in the Region of Augsburg (KORA) S4 study who took part in the KORA Fit follow-up (2018–2019, aged 54–74 years) examination were eligible. Citrate plasma samples were collected in fasted participants. After the exclusion of participants with anticoagulative treatment, 776 participants (420 women and 356 men) with analytic data on hemostatic factors were included in the present analysis. Linear regression models were used to explore the association between BMI or WC with hemostatic markers, adjusted for sex, age, alcohol consumption, education, smoking status, and physical activity. In a second model, additional adjustments were made for the prevalence of stroke, hypertension, myocardial infarction, serum non-HDL cholesterol, and serum triglycerides. *Results*: In the multivariable models (with or without health conditions), significant positive associations with BMI were obtained for plasma concentrations of D-dimers, factor VIII, fibrinogen D, protein S, and quick value, while INR and antithrombin III were inversely associated. Similar to BMI, WC was significantly associated with all hemostatic factors, except for aPTT. *Conclusion*: In this population-based study, both increasing BMI and WC affect the blood coagulation system. Thus, modification of a prothrombotic coagulation profile emerged as a potential target for primary prevention in obese subjects.

## 1. Introduction

The WHO declared obesity an epidemic as it became a worldwide health threat affecting people of all ages, sex, and various socioeconomic status [1]. According to the WHO, between 1975 and 2016 the number of obese individuals nearly tripled [2]. Due to obesity being associated with many diseases such as type-2 diabetes, cardiovascular diseases, or cancers of different sites, the consequences of this development are important for health and the economy [3]. The factors contributing to obesity are manifold. Eventually, a positive energy balance results in the storage of fat in adipocytes and other cells, such as hepatocytes. Physical activity and dietary behaviour, as well as genetics and physiology, have a strong impact on excess body fat accumulation [4]. It is well known that the effect of obesity on atherosclerosis is mediated by chronic inflammation, hyperlipidemia, and endothelial dysfunction with the formation of foam cells and atherosclerotic plaques [5,6]. Also, the effects of obesity on the risk of thrombosis due to an impaired hemostatic balance leading to procoagulant and hypofibrinolytic states is well known [7]. So far, the impact of obesity on coagulation factors has been mainly investigated in patient groups [8]. It is, however, unclear whether such associations also exist at the population level which is the level for primary prevention.

Thus, the present study investigated whether Body Mass Index (BMI), a measure of relative body weight also used to classify overweight and obesity, is related to parameters of the blood coagulation system. In addition, waist circumference (WC) as a measure of visceral body fat accumulation was examined. For the analyses, data from the population-based KORA (Cooperative Health Research in the Region of Augsburg) Fit study were utilized.

## 2. Materials and Methods

### 2.1. Study Sample

KORA is a research platform established in 1996 for population-based surveys. It is the continuation and development of the MONICA (monitoring trends and determinants in cardiovascular disease) studies, which were conducted in the study region of Augsburg from 1984 to 1995 [9]. The MONICA/KORA project comprises four cross-sectional surveys S1 to S4 (S1 1984/85, S2 1989/90, S3 1994/95, and S4 1999/2001) and assessed the health status of the population in the city of Augsburg and the surrounding districts with an emphasis on cardiovascular diseases, lung diseases and diabetes, as well as environmental exposure factors [9].

The KORA S1–S4 studies consist of around 18,000 participants and include a random sample of the inhabitants of the study region between the ages of 25 and 74 years. Since recruitment, all participants are being followed up by questionnaires to assess incident diseases and changes in exposure factors. In the KORA Fit follow-up study (2018–2019), MONICA/KORA study participants born between 1945 to 1964 were re-invited to the study centre for interviews and physical examination. For the present analysis, all participants of KORA S4 who took part in KORA Fit and for which citrate plasma could be collected were considered, leaving 805 study participants. After the exclusion of those with anticoagulative treatment, 776 participants (420 women and 356 men) with analytic data on hemostatic parameters measured in citrate plasma were included in the present analysis. All study participants gave written informed consent. The study protocol was approved by the Ethics Committee of the Bavarian Chamber of Physicians and conducted according to data protection requirements. The investigations were conducted in accordance with the Declaration of Helsinki.

### 2.2. Data Collection

The KORA Fit study consists of an interview and self-administered questionnaires, on, for example, health conditions, and physical measurements.

Anthropometric measurements were performed with the subjects in light clothing and without shoes, according to the World Health Organization MONICA protocol [10]. Body Mass Index was calculated as weight in kg (measured to the nearest 0.1 kg) divided by height in square meters (measured to the nearest 0.5 cm). BMI was categorized according to WHO guidelines as underweight (BMI < 18.5 kg/m^2^), normal weight (BMI ≥ 18.5–<25 kg/m^2^), overweight (BMI ≥ 25–<30 kg/m^2^), and obese (BMI ≥ 30 kg/m^2^) [2,11]. Waist circumference was measured to the nearest 0.1 cm at the midpoint between the lower margin of the least palpable rib and the top of the iliac crest using stretch-resistant tapes [12]. Habitual consumption of alcoholic beverages was assessed for the previous workday and the previous weekend. Alcohol consumption was calculated in grams per day. Self-reported information on prevalent type-2 diabetes, myocardial infarction, and stroke was verified against medical records. Arterial hypertension was defined as systolic blood pressure of ≥140 mm HG, diastolic blood pressure of ≥90 mm HG or known hypertension with the use of antihypertensive drugs. Education years were divided into two groups: less or equal to 12 years and more than 12 years of education. In addition, the smoking habits of participants were classified as current smokers (including irregular smokers), former smokers, and never smokers. Physical activity was categorized into four groups: participation in physical activity for more than two hours per week, at least one hour per week, less than one hour per week, and (almost) no physical activity. Non-HDL cholesterol was calculated by subtracting HDL cholesterol from total cholesterol.

Detailed information on the data collection procedures and examinations in the KORA studies has been described elsewhere [13].

### 2.3. Laboratory Measurements

Hemostatic factors were analyzed in citrate plasma samples collected after an overnight fast. Plasma samples were centrifuged (10 min. at 15°) immediately after collection and then aliquoted and stored at −80 °C. The separation of plasma from the other blood components was completed after 30 min. at the latest. The following hemostatic factors were analyzed: antithrombin III, D-dimers, factor VIII, fibrinogen, protein S, partial thromboplastin time (aPTT), quick value, and international normalized ratio (INR).

Antithrombin III (reference value: 83–118%) was determined by chromogenic activity assay (Innovance Antithrombin, SCS cleaner, Siemens Healthcare Diagnostics, Eschborn, Germany). D-dimers (reference value: <500 µg/dL) were measured by means of a particle-enhanced immunoturbidimetric assay (Innovance D-Dimer Kit, Siemens Healthcare Diagnostics). Factor VIII activity (reference value: 70–150%) was measured photometrically (coagulation factor VIII deficient plasma, Pathromtin SL, CaCl2, Siemens Healthcare Diagnostics), as well as quick value (reference value: 82–125%; Thromborel S, Siemens Healthcare Diagnostics), aPTT (reference value: 26–36 s; Pathromtin SL, CaCl Lösung, Actin FS, Siemens Healthcare Diagnostics), and protein S (reference value: men: 73–130%, women: 52–126%; Hemoclot protein S, OVB-Puffer, CaCl_2_, SCS Cleaner). Fibrinogen (reference value: 210–400 mg/dL) was measured photometrically and turbidimetrically (Multifibren U, Siemens Healthcare Diagnostics). INR (reference value: 0.9–1.15) was calculated from the prothrombin ratio (Thromborel S, Siemens Healthcare Diagnostics) by dividing the thromboplastin time of the subject by that of normal plasma squared with International Sensitivity Index (ISI) as defined by the World Health Organization [14]. Reference values of all hemostatic factors were taken from University Clinic Augsburg, while all other serum parameters were measured at the clinical laboratory of the University Hospital (Klinikum Großhadern) of the Ludwig-Maximilians University in Munich. Measurement procedures were performed and controlled by trained laboratory personnel according to standardized protocols.

### 2.4. Statistical Analysis

For data analysis, the statistical software SPSS, version 26 (IBM Inc., Armonk, New York, USA) was used. The Kolmogorov–Smirnov and Shapiro-Wilk tests were used to test for the normal distribution of data. Normally distributed variables were presented as arithmetic mean and standard deviation. None of the blood coagulation markers were normally distributed and thus described by the median and interquartile range (25–75% percentile). Non-parametric tests were used to test for differences between two (Mann–Whitney U) or more (Kruskal–Wallis) independent samples. A *p*-value of <0.05 was regarded as statistically significant.

Linear regression models with BMI or WC as independent variables were created. The models included the blood coagulation markers described above as dependent variables. The models were first adjusted for age (continuous), alcohol consumption (continuous), sex (male; female), education (<12 years; ≥12 years), smoking status (never; former; current), physical activity (physical activity for more than two hours per week, at least one hour per week, less than one hour per week, and (almost) no physical activity), and in a second model further adjusted for prevalent stroke (yes/no), hypertension (yes/no), myocardial infarction (yes/no), serum non-HDL cholesterol, and triglycerides (continuous).

## 3. Results

The baseline characteristics of the study population are given in Table 1. The mean BMI was higher in men with 28.40 ± 4.23 kg/m^2^ as compared to women with 27.39 ± 5.18 kg/m^2^, leaving more men than women in the overweight and obese categories.

The median concentrations of hemostatic parameters differed between men and women, except for D-dimers, factor VIII, and fibrinogen (Table 2). Median concentrations of all markers were in a normal range, except for protein S.

Table 3 describes the median plasma concentrations of coagulation markers by BMI categories (Table 3). Obese subjects showed the highest concentrations of D-dimers, factor VIII, fibrinogen, and protein S, while antithrombin III concentrations were the lowest. Whereas all other coagulation factors showed no deviation from the normal range, median protein S concentration in obese subjects was outside the normal range (median: 131.70; interquartile range: 113.20; 148.80).

The results of the linear regression models that explored the association between BMI and each of the blood coagulation factors are shown in Table 4. All analyses were adjusted for sex, age, alcohol consumption, education, smoking status, and physical activity (Model 1). In a second model, additional adjustments were made for the prevalence of stroke, hypertension, and myocardial infarction, as well as for plasma non-HDL cholesterol and plasma triglycerides (Model 2). In Model 1, statistically significant positive associations were obtained for plasma concentrations of D-dimers, factor VIII, fibrinogen D, protein S, and quick value, while INR and antithrombin III were inversely associated. The results were slightly attenuated after additional adjustments for health conditions (Model 2). Only aPTT was not significantly related to BMI. Similar associations were seen in linear regression models with WC as the independent variable (Table 5).

## 4. Discussion

This population-based study identified significant associations between nearly all analyzed blood coagulation markers and BMI as well as WC. Increased BMI or WC were related to higher concentrations of D-dimers, factor VIII, fibrinogen, protein S, and quick value and decreased values of antithrombin III and INR.

Many studies confirmed a higher risk of early mortality for people with a high body fat mass (often expressed as BMI) [15] but data on hemostatic factors and mortality is rare. Ageno and co-workers [16] searched databases from 1996 to 2006 and found 15 studies of sufficient quality to summarize the evidence for the link between obesity and venous thromboembolism. Eight case-control studies and one cohort study were combined in a meta-analysis; the pooled relative risk of venous thromboembolism was 2.33 (95% CI: 1.68–2.34) in obese subjects versus subjects with normal body weight.

The basis for the correct interpretation of our findings is the physiologic role each hemostatic factor has in the complex blood coagulation system. D-dimers reflect both activations of fibrinolysis and thrombin production [17]. It is clinically used to indicate hypercoagulability in various disorders like venous thromboembolism, ischemic heart disease, trauma, and infection [18,19]. Coagulation factor VIII is a glycoprotein synthesized mainly in hepatocytes, but also in kidneys, endothelial cells, and lymphatic tissue [20]. Higher plasma concentrations of both factors indicate thrombotic events, and lower levels eliminate the risk of thrombolytic events. Another glycoprotein produced in the liver is fibrinogen. Fibrinogen gets converted enzymatically by thrombin to fibrin and contributes to the formation of blood clots, in reaction to vascular or tissue injury [21]. Protein S, a co-factor of protein C, is created in the liver and influences blood coagulation by increasing fibrinolysis; it is dependent on vitamin K. Low levels of protein S lead to a higher risk of thrombophilia, which itself is associated with a higher occurrence of thrombosis. Differences in protein S levels can be due to low vitamin K levels, pregnancy, or several chronic diseases [22]. Antithrombin III is a glycoprotein belonging to the group of serine protease inhibitors. By inhibiting several factors of the coagulation system, especially thrombin, and factors VIIa, IXa, Xa, Xia, and XIIa, antithrombin III holds a central role in regulating the coagulation cascade [23]. Low levels of antithrombin III lead to a very high risk of thrombosis [24] and can also suggest reduced synthesis and underlying liver disease [25]. Antithrombin III is a physiologic anticoagulant and its physiologic effects are increased by a factor of 1000 in the presence of heparin.

A few other investigations have also confirmed positive associations of coagulation markers with rising BMI as observed in our study. Hörber et al. [26] investigated the relationship between obesity, in particular, body fat distribution, and hemostatic parameters. They used data from 150 subjects with impaired glucose tolerance and/or impaired fasting glucose, which were randomly selected from the “Prediabetes Lifestyle Intervention Study” (PLIS). The participants were exclusively recruited at the University Hospital in Tübingen. Regression models were not fully adjusted for possible confounders except age and sex. They reported positive associations between BMI and factor VIII, protein S, and D-dimers, while antithrombin III was inversely associated with BMI. These findings are in line with our results in a middle-aged population. However, the literature also reports positive associations of antithrombin III levels with rising BMI [27]. Christensen et al. [28] described the influence of BMI on the rise of factor VIII through adipose tissue influencing hepatic metabolism and the production of hemostatic factors in the liver.

Clotting tests used in clinical practice to describe failures in the intrinsic or extrinsic pathway are aPTT, INR, and quick value. They mostly depend on partial thromboplastin time. Quick and INR are inversely proportional to each other, meaning that a rise in one causes a decrease in the other. This was also confirmed in the present study. While Quick and INR detect influences on extrinsic blood coagulation, aPTT detects those in the intrinsic system. To the best of our knowledge, the influence of BMI or WC on the results of clotting tests has not been reported so far.

Since, in our study, BMI- and WC-based analyses indicated similar associations with markers of coagulation, it appears to be the extent of body fat in general—and not limited to visceral fat—that affects the coagulation system. In a review article, De Pergola et al. [29] described coagulation and fibrinolysis abnormalities in obese patients who showed higher plasma concentrations of all prothrombotic factors, especially fibrinogen, compared to non-obese, and with a positive association with central fatty tissue accumulation. Their review also confirms the great influence of plasminogen activator inhibitor (PAI-1) on the risk of coronary events and atherothrombosis. With PAI-1 being the primary physiological inhibitor of plasminogen activation, it has a significant effect on fibrinolysis [30]. Several more studies cited in this review showed that plasma levels of PAI-1 in obese people directly correlate with visceral fat independent of metabolic and non-metabolic variables. In a sub-study of the Prediabetes Lifestyle Intervention Study, Hörber et al. [31] examined the effect of lifestyle intervention on hemostatic factors. They showed that lifestyle intervention can improve prothrombotic state and decrease several coagulation factors, among others, protein S, factor VIII, and PAI-1. They used data from 100 individuals with impaired glucose tolerance or impaired fasting blood glucose, who participated in a one-year lifestyle intervention, including precise metabolic phenotyping, MR-based determination of liver fat content, and analysis of coagulation parameters before and after this intervention. The results show a potential way to improve prothrombotic status through lifestyle intervention and provide insight into possible future treatment targets.

The strengths of our study are a large sample size, the population-based design, the availability of standardized data on cardiovascular risk factors, and standardized anthropometric and laboratory measurements. However, several limitations of the study also need to be considered. The lack of measurement of other important coagulation factors like thrombin, von Willebrand factor, and plasminogen activator inhibitor limits the significance of the study. Furthermore, follow-up studies need to consider the distribution of subcutaneous fat and visceral fat by bioelectrical impedance analysis (BIA) dual-energy X-ray absorptiometry (DEXA), or magnetic resonance imaging (MRI) measurements. Finally, the results may not be transferable to persons of other ethnicity or other ages, since only participants in the Augsburg region ranging in age from 53 to 74 years were considered.

## 5. Conclusions

Overall, our results in this population-based study indicated that an increase in BMI and WC impacts the blood coagulation system in a sense that the balance between coagulation and fibrinolysis is shifted towards a more prothrombotic constellation, which is confirmed by the increase in pro-coagulative factors and the result of clotting tests in our study. As an additional mechanism to explain the effect of obesity on the development of selected chronic diseases, the coagulation system could be considered a target for the primary prevention of thrombotic events.

## Figures and Tables

**Table 1:** Characteristics of the study participants ^a^; overall and by sex.

		Total (n = 776)		Males (n = 356)		Females (n = 420)	
				Mean	SD		
Age [years]		63	6	63	6	63	6
BMI [kg/m²]		27.86	4.79	28.40	4.23	27.39	5.18
Waist circumference [cm]		94.0	13.8	100.5	12.2	88.4	12.6
		Median (25th–75th percentile)					
Alcohol consumption ^b^ [g/d]		5.71 (0.00; 22.86)		5.71 (0.00; 21.50)		6.70 (0.00; 22.86)	
				N	%		
BMI [kg/m²]	Underweight	5	0.6%	0	0%	5	1.0%
	Normal weight	229	29.5%	80	22.5%	149	35.5%
	Overweight	307	39.6%	156	43.8%	151	36.0%
	Obese	235	30.3%	120	33.7%	115	27.4%
Education [years]	≤12 years	471	60.7%	198	56.0%	273	63.0%
	>12 years	305	39.3%	158	44.0%	147	37.0%
Physical activity	≥2 h/week	284	36.6%	133	37.5%	151	36.0%
	1 h/week	264	34.0%	115	32.5%	149	35.5%
	<1 h/week	94	12.1%	50	14.0%	44	10.5%
	(almost) no activity	134	17.3%	58	16.0%	76	18.0%
Smoking	Current smoker	106	13.6%	53	15.0%	53	13.0%
	Former smoker	335	43.2%	176	49.5%	159	38.0%
	Never smoker	335	43.2%	127	35.5%	208	49.0%
Hypertension ^c^	Yes	361	46.5%	196	55.0%	165	39.0%
	No	414	53.5%	159	45.0%	255	61.0%
Myocardial infarction	Yes	22	2.8%	18	5.0%	4	1.0%
	No	754	97.2%	338	95.0%	416	99.0%
Stroke	Yes	18	2.3%	12	3.4%	6	1.5%
	No	758	97.7%	344	96.6%	414	98.5%
Type-2 diabetes ^c^	Yes	64	8.3%	32	9.0%	32	7.6%
	No	710	91.7%	324	91.0%	386	92.4%

^a^ After exclusion of individuals with anticoagulation therapy (n = 29); ^b^ n = 775; ^c^ n = 775.

**Table 2:** Plasma concentrations of blood coagulation factors in all participants and by sex ^a,d^.

	Total	Males	Females	*p*-Value ^d^
	Median (25th–75th percentile)			
Antithrombin III [mg/dL]	102.30 (95.50; 108.90)	98.80 (93.30; 105.55)	104.70 (98.30; 110.60)	0.001
D-dimers [µg/L]	407 (309; 556)	408 (316; 563)	406 (306; 554)	0.642
Faktor VIII [%]	121.70 (97.40; 143.00)	119.80 (94.90; 141.80)	123.75 (100.90; 143.55)	0.181
Fibrinogen D [mg/dL]	294.55 (260.40; 334.20)	288.00 (257.50; 326.70)	299.55 (263.25; 339.75)	0.041
aPTT [s] ^b^	30.70 (28.70; 32.90)	31.15 (29.30; 33.50)	30.25 (28.70; 32.90)	0.001
Protein S [%]	125.70 (105.45; 146.10)	131.70 (111.00; 153.90)	120.00 (101.30; 114.50)	0.001
Quick value [%]	108.80 (102.20; 114.50)	106.70 (100.20; 112.50)	110.35 (104.50; 115.70)	0.001
INR ^c^	0.95 (0.92; 0.99)	0.97 (0.93; 1.01)	0.95 (0.91; 0.98)	0.001

^a^ After exclusion of individuals with anticoagulation therapy (n = 29); ^b^ activated partial thromboplastin time; ^c^ international normalized ratio; ^d^ Mann–Whitney U Test *p* < 0.05.

**Table 3:** Plasma concentrations of hemostatic factors ^a^ (median, IQR) according to Body Mass Index (BMI) categories.

BMI [kg/m^2^]	Underweight [<18.5]	Normal Weight [≥18.5–<25]	Overweight [≥25–<30]	Obesity [≥30]	*p*-Value ^d^
	(n = 5)	(n = 229)	(n = 307)	(n = 235)	
		Median (25th–75th percentile)			
Antithrombin III [mg/dL]	104.7 (94.0; 112.0)	106.5 (99.5; 113.10)	100.60 (94.70; 107.30)	99.90 (93.40; 106.10)	0.001
D-dimers [µg/L]	335 (289; 476)	368 (276; 503)	392 (308; 552)	449 (348; 691)	0.001
Faktor VIII [%]	112.40 (100.30; 125.30)	119.80 (9740; 140.35)	116.00 (93.70; 137.90)	132.50 (107.30; 154.30)	0.001
Fibrinogen D [mg/dL]	306.20 (296.10; 336.10)	278.60 (247.10; 316.20)	288.30 (261.90; 325.30)	317.70 (279.10; 357.30)	0.001
aPTT [s] ^b^	28.80 (28.10; 30.30)	30.60 (28.60; 32.90)	31.10 (29.1; 33.30)	30.40 (28.40; 32.70)	0.070
Protein S [%]	128.50 (98.90; 154.50)	115.50 (98.80; 133.20)	129.25 (105.70; 148.80)	131.70 (113.20; 148.80)	0.001
Quick value [%]	111.40 (107.80; 121.00)	108.05 (100.45; 113.70)	108.00 (101.7; 113.90)	110.20 (104.25; 115.90)	0.004
INR ^c^	0.94 (0.89; 0.96)	0.96 (0.93; 1.00)	0.96 (0.92; 1.00)	0.95 (0.91; 0.98)	0.004

^a^ After exclusion of individuals with anticoagulation therapy (n = 29); ^b^ activated partial thromboplastin time; ^c^ international normalized ratio; ^d^ Kruskal–Wallis test *p* < 0.05.

**Table 4:** Association between Body Mass Index and blood coagulation parameters ^a^.

	Model 1 ^b^			Model 2 ^c^		
	β Estimate	95% Confidence Interval	*p*-Value	β Estimate	95% Confidence Interval	*p*-Value
Antithrombin III [mg/dL]	−0.435	−0.592; −0.279	<0.001	−0.468	−0.633; −0.302	<0.001
Ln D-dimers	0.020	0.013; 0.028	<0.001	0.020	0.012; 0.028	<0.001
Faktor VIII [%]	1.273	0.740; 1.806	<0.001	1.145	0.581; 1.710	<0.001
Fibrinogen D [mg/dL]	2.987	2.045; 3.929	<0.001	2.923	1.921; 3.926	<0.001
aPTT [s] ^d^	−0.036	−0.087; 0.014	0.160	−0.043	−0.097; 0.010	0.114
Protein S [%]	0.943	0.425; 1.460	<0.001	0.609	0.070; 1.147	0.027
Quick value [%]	0.214	0.070; 0.358	0.004	0.177	0.026; 0.328	0.021
INR ^e^	−0.001	−0.002; 0.000	0.005	−0.001	−0.002; 0.000	0.022

^a^ After exclusion of individuals with anticoagulation therapy (n = 29); ^b^ dependent variables: blood coagulation markers, independent variable: Body Mass Index, adjusted for age, alcohol consumption, sex, education, smoking, and physical activity; ^c^ dependent variables: blood coagulation markers, independent variable: Body Mass Index, additionally adjusted for stroke, hypertension, myocardial infarction, non-HDL cholesterol, triglycerides; ^d^ activated partial thromboplastin time; ^e^ international normalized ratio.

**Table 5:** Association between waist circumference and blood coagulation parameters ^a^.

	Model 1 ^b^			Model 2 ^c^		
	β Estimate	95% Confidence Interval	*p*-Value	β Estimate	95% Confidence Interval	*p*-Value
Antithrombin III [mg/dL]	−0.180	−0.241; −0.118	<0.001	−0.191	−0.256; −0.126	<0.001
Ln D-dimers	0.008	0.005; 0.011	<0.001	0.008	0.005; 0.011	<0.001
Faktor VIII [%]	0.441	0.231; 0.651	<0.001	0.385	0.162; 0.608	0.001
Fibrinogen D [mg/dL]	1.035	0.664; 1.406	<0.001	1.021	0.623; 1.418	<0.001
aPTT [s] ^d^	−0.016	−0.036; 0.004	0.114	−0.020	−0.041; 0.001	0.064
Protein S [%]	0.395	0.193; 0.597	<0.001	0.265	0.054; 0.477	0.014
Quick value [%]	0.098	0.042; 0.154	0.001	0.086	0.026; 0.146	0.005
INR ^e^	−0.001	−0.001; 0.000	0.001	−0.001	−0.001; 0.000	0.005

^a^ After exclusion of individuals with anticoagulation therapy (n = 29); ^b^ dependent variables: Blood coagulation markers, independent variable: Body Mass Index, adjusted for age, alcohol consumption, sex, education, smoking, and physical activity; ^c^ dependent variables: blood coagulation markers, independent variable: Body Mass Index, additionally adjusted for stroke, hypertension, myocardial infarction, non-HDL cholesterol, triglycerides; ^d^ activated partial thromboplastin time; ^e^ international normalized ratio.

## Data Availability

The data are subject to national data protection laws, and restrictions were imposed by the Ethics Committee of the Bavarian Chamber of Physicians to ensure data privacy of the study participants. Therefore, data cannot be made freely available in a public repository. However, data can be requested through an individual project agreement with KORA via the online portal KORA (https://www.helmholtz-munich.de/en/epi. Accessed on 25 January 2023).
